# Estimating Shape and Micro-Motion Parameter of Rotationally Symmetric Space Objects from the Infrared Signature

**DOI:** 10.3390/s16101722

**Published:** 2016-10-17

**Authors:** Yabei Wu, Huanzhang Lu, Fei Zhao, Zhiyong Zhang

**Affiliations:** Automatic Target Recognition Laboratory, National University of Defense Technology, Deya Road, Changsha 410073, China; luhz@nudt.edu.cn (H.L.); f_z2010@126.com (F.Z.); nudtzzy@163.com (Z.Z.)

**Keywords:** space objects, infrared signature, projecting feature, micro-motion

## Abstract

Shape serves as an important additional feature for space target classification, which is complementary to those made available. Since different shapes lead to different projection functions, the projection property can be regarded as one kind of shape feature. In this work, the problem of estimating the projection function from the infrared signature of the object is addressed. We show that the projection function of any rotationally symmetric object can be approximately represented as a linear combination of some base functions. Based on this fact, the signal model of the emissivity-area product sequence is constructed, which is a particular mathematical function of the linear coefficients and micro-motion parameters. Then, the least square estimator is proposed to estimate the projection function and micro-motion parameters jointly. Experiments validate the effectiveness of the proposed method.

## 1. Introduction

Exo-atmosphere object discrimination is a key technology for precise guidance systems and satellite remote sensing systems. Application can be found in the research of ballistic warheads, decoys, etc. [1,2].

The performance of the discriminating system is heavily dependent on the choice of data representation (or features) on which they are applied. Numerous features have been extracted in the past few decades. The micro-motion features, which reflect the unique dynamic and structural characteristics of the target, serve as important features for target recognition and classification [3,4,5]. The time-frequency representation based methods have been used to extract the micro-motion parameter for maneuvering space objects among the radar community [6,7,8]. Other than using the radar data, the discriminating system based on the IR (infrared) camera is also an important research field [9,10]. Commonly, due to the long observing range (e.g., 100 km), even relatively large objects are represented as single pixels on the IR image [11]. The research on IR dim target detection and tracking provides the positions of the point target in the IR image time series [12,13]. Then, the IR signature of the point object, i.e., the infrared radiation intensity time series, can be extracted. Object discrimination based on the IR signature has been a hot topic in recent years. Since different objects may possess different temperatures and cool at varying rates, the features about temperature are extracted from the IR signature and used extensively. Based on the fact that the object temperature can be approximately estimated using the radiation ratio of two different wavelengths [14], Liu et al. propose a classifying system using the mean radiation ratio of two wavelength bands and the varying range of the ratio during the observing interval as features [15]. Wang et al. use the object temperature estimated from the multi-band sensor data for object classification [16]. Other than the micro-motion and temperature features, shape also serves as an important additional feature for target recognition and classification that is complementary to those made available by existing methods. For example, the reentry vehicle may be a cone and the attitude control module is a truncated cone [17]. However, to the best of our knowledge, there is no work on extracting shape features from the IR signature.

In this work, we show that there is potential to extract shape information from the IR signature. After the temperature of the object has been estimated, measurements of the emissivity-area product can be acquired whose value is proportional to the projection area of the object at line of sight [16,18]. Given the shape, the projection area of the object is just a function of the observing angle. Since different shapes lead to varying projection functions, the projection function of the object can be regarded as the shape feature. We show that the projection function of any object with rotationally symmetric shape can be represented as the linear combination of the projection functions of truncated cones with varying half cone angles. Based on this projection function representation, the signal model of the emissivity-area product time sequence is constructed that is a particular mathematical function of the linear projection coefficients and micro-motion parameters. Then, the least square error estimator is proposed to estimate the projection function and micro-motion parameters jointly. Experiments show the effectiveness of the proposed algorithm.

The rest of this paper is organized as follows. We first present the signal model of the emissivity-area measurements in Section 2. The parameter estimating algorithm is presented in Section 3, followed by the experiments in Section 4. Concluding remarks are provided in the last section.

## 2. Signal Model

For an object at an absolute temperature *T*, the total power $P_{S}\left(\lambda ,T\right)$ received by the sensor in a small bandwidth Δλ centered at wavelength *λ* is approximately given by [16]

(1) $$P_{S}\left(\lambda ,T\right)=\frac{\tau \left(\lambda \right)A_{O}}{\pi R^{2}}A_{C}\varepsilon \left(\lambda ,T\right)E_{b}\left(\lambda ,T\right)\Delta \lambda$$

where τ(λ) is the spectral optical transmittance, $A_{O}$ is the receiving area of the sensor optics, *R* is the range of object to the aperture of a system, and $A_{C}$ denotes the projection area of the object at the line of sight. ε(λ,T) is the emissivity of the surface material of the object and $E_{b}\left(\lambda ,T\right)$ denotes the spectral radiant exitance of blackbody, defined by Planck’s radiation law. By comparing the power of the target signal in several infrared wavelength bands, we will be able to perform a fit to the Planck blackbody curve to obtain an estimate of the target temperature (a temperature obtained this way is known as a color or distribution temperature). Once we estimate the temperature of an object, it is also possible to estimate the emissivity-area product as a function of measured or estimated values [16,18]. This is given by

(2) $$Y=\varepsilon \left(\lambda ,T\right)A_{C}=\frac{\pi R^{2}P_{S}\left(\lambda ,T\right)}{\tau \left(\lambda \right)A_{O}E_{b}\left(\lambda ,T\right)\Delta \lambda }$$

Commonly, the space object is approximated to be a graybody in the given wavelength interval, i.e., ε(λ,T)=ε [19]. Thus, the changing of emissivity-area product is mainly caused by the changing of the projection area $A_{C}$. The changing of $A_{C}$ is correlated with the shape and micro-motion of the object. The rotationally symmetric object constitutes one of the typical classes of object in the ballistic target complex. For example, the warhead and the decoy may be cones. The attitude control model is a cylinder, and coning is the way of micro-motion for the target. Due to the fact that different objects possess different shapes and micro-motion dynamics, extracting the shape and micro-motion information is important for recognizing the lethal object in the target complex, and this problem is addressed in this work. First, the projection variation of the coning rotationally symmetric object is addressed.

### 2.1. The Projection of a Rotationally Symmetric Object

**Figure 1:**
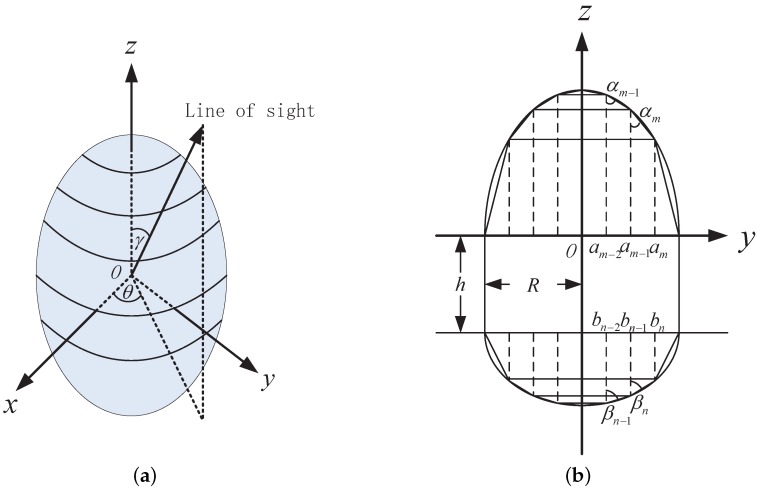
Illustration of the mathematical representation for the projection function. (**a**) shows the projection geometry and the partition of the surface into many rings; and (**b**) shows the approximation of the ring surface with the truncated cone surface in the side view.

To model the variation of the projection area for a rotationally symmetric object, the first step is to model the projection function, i.e., the relation between the projection area and observing angle. Figure 1 shows the idea of the projection function representation in this work. As shown in Figure 1a, in the local coordinate system, the symmetric axis of the object attaches to the *z*-axis. The surface of the object is partitioned into many rings by planes parallel to the *x* – *y* plane. In Figure 1b, the surface of each ring is approximated with a truncated cone. $\alpha_{m},m=1,\cdots ,M$ and $\beta_{n},n=1,\cdots ,N$ denote the half cone angle of these truncated cones. When *M* and *N* are sufficiently large, the approximating error is negligible. As the projection of the object is the summation of the projection of each ring, it can be approximated with the summation of the projection of each truncated cone. In Figure 1a, *γ* is the elevation angle of the line of sight and *θ* is the azimuth angle. For the rotationally symmetric object or surface, its projection at the line of sight is only related with the elevation angle *γ*. Changing *θ* does not change the projection area. Thus, the projection of the rotationally symmetric object can be approximately represented as

(3) $$A_{C}\left(\gamma \right)=\sum_{m=1}^{M}A_{m}\left(\gamma \right)+\sum_{n=1}^{N}B_{n}\left(\gamma \right)+C_{0}\left(\gamma \right)$$

where $A_{m}\left(\gamma \right)$ is the projection of the top truncated cone indexed by *m* (top means that the direction of the cone is (0,0,1) in the local coordinate system), $B_{n}\left(\gamma \right)$ is the projection of the bottom truncated cone indexed by *n* (bottom means the cone directs at (0,0,−1) in the local coordinate system), and $C_{0}\left(\gamma \right)$ is the projection of the medium cylinder surface part. After computing the projection function of the top truncated cone, we get

(4) $$A_{m}\left(\gamma \right)=\pi \left(a_{m}^{2}-a_{m-1}^{2}\right)A\left(\cot\alpha_{m},\gamma \right)$$

(5) $$A\left(\cot\alpha ,\gamma \right)=\left\{\begin{array}{cc} 0, & \cos\gamma \in [-1,-\cos\alpha ]\\ \frac{1}{\pi }\left[\cos\gamma \cdot \arccos\left(-\frac{\cot\gamma }{\cot\alpha }\right)+\sin\gamma \sqrt{\cot^{2}\alpha -\cot^{2}\gamma }\right], & \cos\gamma \in (-\cos\alpha ,\cos\alpha )\\ \cos\gamma , & \cos\gamma \in [\cos\alpha ,1] \end{array}\right.$$

where $a_{m}$ denotes the outside radius of the truncated cone *m* while $a_{m-1}$ is its inside radius, $a_{0}=0,a_{M}=R$, and *R* is the radius of the medium cylinder surface. $\pi (a_{m}^{2}-a_{m-1}^{2})$ denotes *x*–*y* plane projection of the truncated cone *m*. A(cotα,γ) denotes the projection of the top truncated cone with the half cone angle *α* and unit *x*–*y* plane projection:

(6) $$B_{n}\left(\gamma \right)=\pi \left(b_{n}^{2}-b_{n-1}^{2}\right)B\left(\cot\beta_{n},\gamma \right)$$

(7) B(cotα,γ)=A(cotα,π−γ)

where $b_{n}$ denotes the outside radius of the truncated cone *n* while $b_{n-1}$ is the inside radius, $b_{0}=0,b_{M}=R$. $\pi (b_{n}^{2}-b_{n-1}^{2})$ denotes the projection area of the truncated cone *n* on the *x*–*y* plane. B(cotα,γ) denotes the projection area of the bottom truncated cone with the half cone angle *α* and unit projection area on the *x*–*y* plane. For the central cylinder:

(8) $$C_{0}\left(\gamma \right)=2RhC\left(\gamma \right)=2Rh\sqrt{1-\cos^{2}\gamma }$$

where *h* is the height of the medium cylinder surface.

Estimation of the projection function needs proper projection function representation. In order to construct effective projection function representation, we quantify the the half cone angle over the range of (0, (${\cot\alpha )}_{max}$) with an interval of Δ, and then Equation (3) can be approximately represented as

(9) $$A_{C}\left(\gamma \right)=\sum_{i=0}^{K}a_{i}A_{i}\left(\gamma \right)+\sum_{j=0}^{K}b_{j}B_{j}\left(\gamma \right)+c\cdot C\left(\gamma \right),s.t.\sum_{i=0}^{K}a_{i}=\sum_{j=0}^{K}b_{j}=\pi R^{2}$$

where $A_{i}\left(\gamma \right)=A\left(i\Delta ,\gamma \right)$, $B_{j}\left(\gamma \right)=B\left(j\Delta ,\gamma \right)$, and $K\Delta ={\left(\cot\alpha \right)}_{max}$. $a_{i}=\sum_{\left\{m|\cot\;\alpha_{m}\approx i\Delta \right\}}\pi \left(a_{m}^{2}-a_{m-1}^{2}\right)$ is the *x*–*y* plane projection of the top truncated cone with half cone angle iΔ, and $b_{j}=\sum_{\left\{n|\cot\;\beta_{n}\approx j\Delta \right\}}\pi \left(b_{n}^{2}-b_{n-1}^{2}\right)$ is the *x*–*y* plane projection of the bottom truncated cone with half cone angle jΔ. c=2Rh. The restriction indicates that the summation of the *x* – *y* plane projection for all the top truncated cones and all the bottom truncated cones are the same. The smaller the quantization interval Δ, the smaller the approximating error. Quantization enables the linear representation of the projection function for a rotationally symmetric object with a fixed number of known base projection functions, and the projection function estimation is performed by estimating the coefficients. This is the projection function representation proposed in this work.

According to Equation (9), the items in the coefficient vector s = ($a_{0}$,⋯,$a_{K}$, $b_{0}$,⋯,$b_{K}$,*c*) indicate the *x*–*y* plane projection area of the truncated cones. By restricting the shape to concave, we can recover one shape from the coefficients. This process can also be showed with Figure 1. By putting the truncated cone with a bigger half cone angle *α* closer to the *z*-axis, we can compute the inside and outside radius of each truncated cone one by one. As a result, one shape can be constructed. The correspondence between the coefficient and the *x* – *y* plane projection area indicates that every coefficient vector s in the space $R^{2K+3}$ following the restriction in Equation (9) is able to recover one shape. Thus, the coefficient vector can also be regarded as the shape representation for the concave rotationally symmetric object.

**Figure 2:**
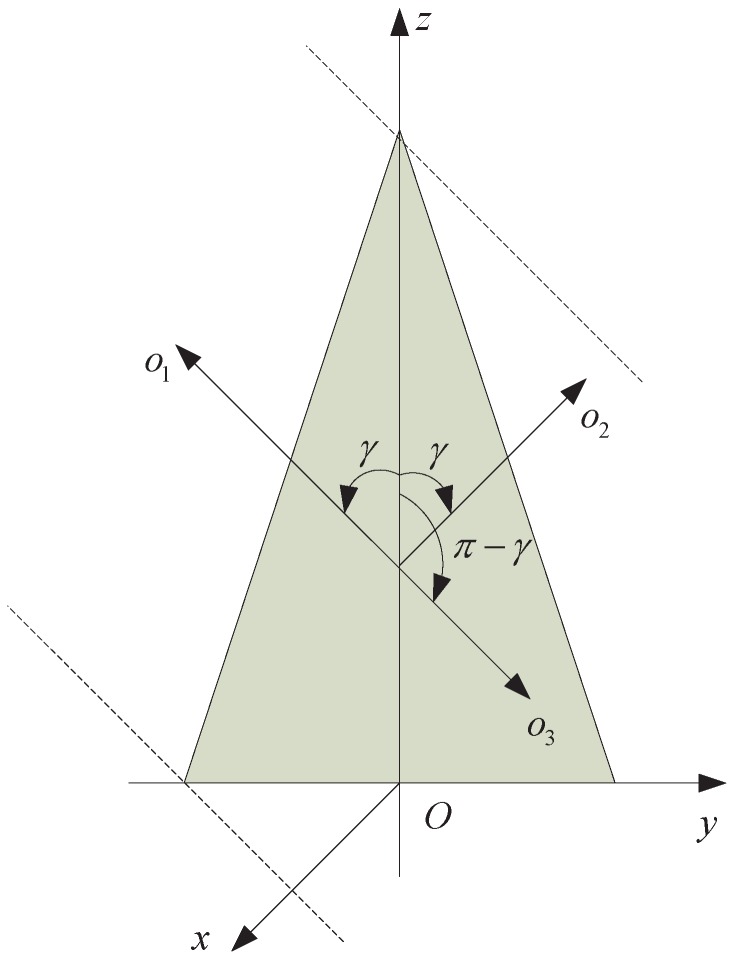
Illustration of the even property of the projection function.

One important property of the projection function $A_{C}\left(\gamma \right)$ for the rotationally symmetric object is that it is an even function of cosγ. It is easy to validate this property. As shown in Figure 2, ${\vec{o}}_{1},{\vec{o}}_{2},{\vec{o}}_{3}$ denote three different lines of sight in the *y* – *z* plane. We can see that $A_{C}\left({\vec{o}}_{1}\right)=A_{C}\left({\vec{o}}_{2}\right)$, $A_{C}\left({\vec{o}}_{1}\right)=A_{C}\left({\vec{o}}_{3}\right)$, and then $A_{C}\left({\vec{o}}_{2}\right)=A_{C}\left({\vec{o}}_{3}\right)$, i.e., $A_{C}\left(\gamma \right)=A_{C}\left(\pi -\gamma \right)$. Thus, $A_{C}\left(\cos\gamma \right)=A_{C}\left(-\cos\gamma \right)$.

**Figure 3:**
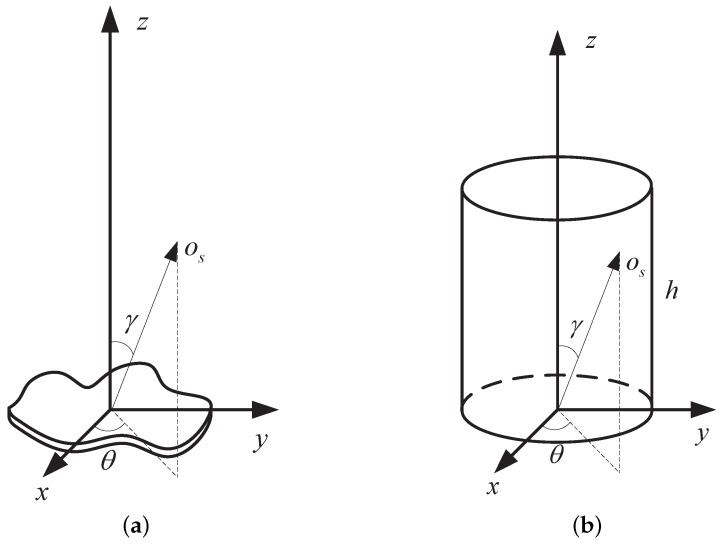

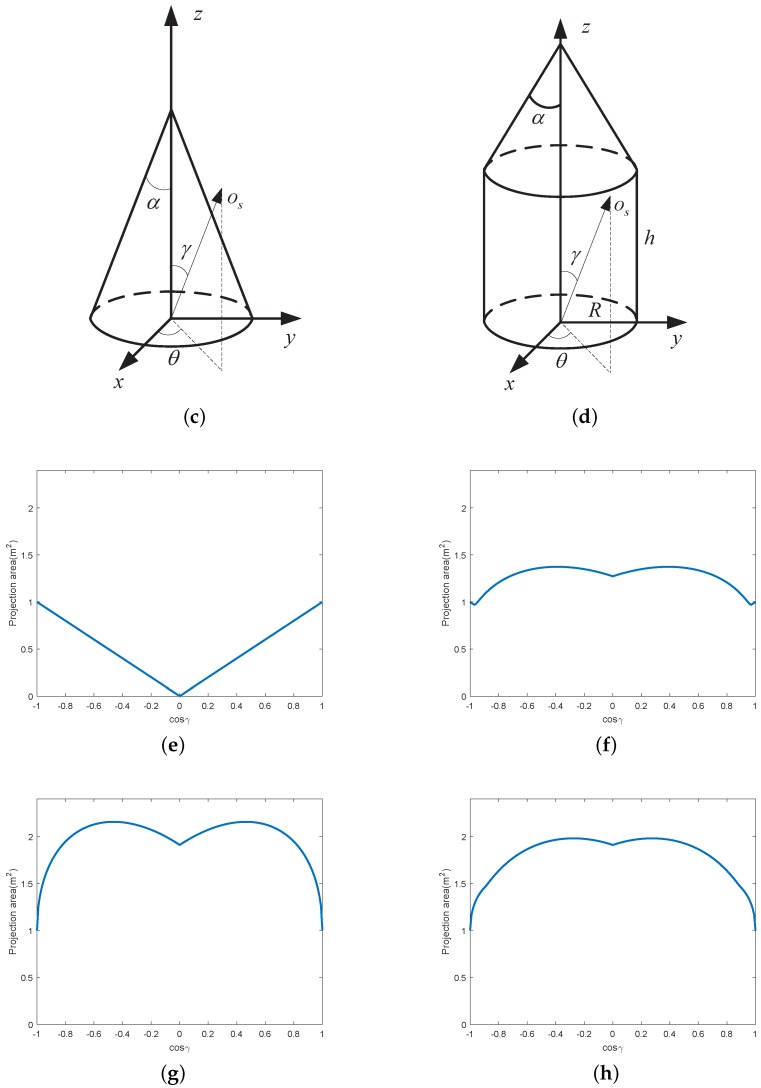
Illustration of the projection function for different shapes. (**a**) shows the projection geometry of the plane; (**b**) shows the projection geometry of the cylinder; (**c**) shows the projection geometry of the cone; (**d**) shows the projection geometry of the cone–cylinder; (**e**) shows the projection function of the plane; (**f**) shows the projection function of the cylinder; (**g**) shows the projection of the cone; and (**h**) shows the projection function of the cone–cylinder.

Based on Equation (3), it is easy to compute the projection function of some typical shapes, e.g., plane, cylinder, cone and cone–cylinder combination. In the ballistic target complex, the debris is usually a plane, the attitude control model is a cylinder and the warhead is a cone or cone–cylinder. As illustrated in Figure 3, the projection geometry of these four shapes are showed in the first row, and their corresponding projection functions are showed in the second row. It is clear that different shapes lead to varying projection functions. Thus, estimating the projection function is useful for discriminating the shape of the object.

### 2.2. The Variation of Observing Angle

**Figure 4:**
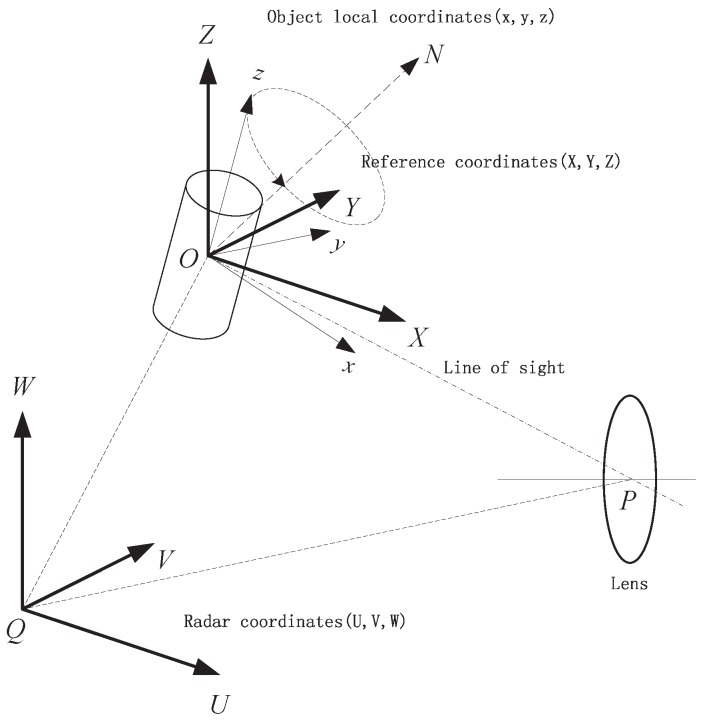
Geometry of radar, sensor and coning object.

Having known that the variation of projection area $A_{C}$ is only related to the observing angle *γ*, the changing of *γ* is addressed in this section. The geometry of the infrared sensor and a target with coning motion is depicted in Figure 4. The radar is located at the origin of the radar coordinate system (U,V,W) and the target’s local coordinate system is (x,y,z), in which the *z*-axis attaches to the symmetric axis of the object. The target has a coning motion along the axis $\vec{ON}$, which intersects with the *z*-axis at the origin point *O* of the local coordinate system. The reference coordinate system (X,Y,Z), which is parallel to the radar coordinates (U,V,W) and shares the same origin *O* with the target local coordinates (x,y,z), has the same initial velocity and acceleration as the target but has no rotation with respect to the radar coordinates. The azimuth and elevation angle of the coning axis $\vec{ON}$ with respect to the reference coordinates (X,Y,Z) are $\alpha_{N}$ and $\beta_{N}$, respectively. Suppose the target has a coning motion with an angular velocity of *ω* rad/s. According to the Rodrigues formula [3], at time *t*, the rotation matrix in the (X,Y,Z) becomes

(10) $$R\left(t\right)=I+K\sin\omega t+K^{2}\left(1-\cos\omega t\right)$$

where the skew symmetric matrix *K* is defined by

(11) $$K=\left[\begin{array}{ccc} 0 & -\sin\beta_{N} & \sin\alpha_{N}\cos\beta_{N}\\ \sin\beta_{N} & 0 & -\cos\alpha_{N}\cos\beta_{N}\\ -\sin\alpha_{N}\cos\beta_{N} & \cos\alpha_{N}\cos\beta_{N} & 0 \end{array}\right]$$

Assume the initial azimuth and elevation angle of the symmetric axis of the object in the reference coordinate system is $\alpha_{0}$ and $\beta_{0}$, and the initial unit vector of the symmetric axis is ${\vec{n}}_{0}={\left[\cos\alpha_{0}\cos\beta_{0},\sin\alpha_{0}\cos\beta_{0},\sin\beta_{0}\right]}^{T}$. Then, at time *t*, the unit direction vector of the symmetric axis will move to

(12) $$\vec{n}\left(t\right)=R\left(t\right){\vec{n}}_{0}$$

The angle *γ* (the angle between the symmetric axis of the object and the line of sight) can be computed as

(13) $$\cos\gamma \left(t\right)=\vec{n}\left(t\right)\frac{\vec{OP}\left(t\right)}{|\vec{OP}\left(t\right)|}=\vec{n}\left(t\right)\cdot \vec{o}\left(t\right)$$

In the radar coordinate system, the position of the target and sensor can be acquired by the ground-based radar system. Then, the unit direction vector $\vec{OP}\left(t\right)$ can be computed. $|\vec{OP}\left(t\right)|$ denotes the length of the vector, which is also the observing distance. $\vec{o}\left(t\right)$ is the unit direction vector of the line of sight.

In summary, Equations (2), (9) and (13) form the mathematical model of the emissivity-area product time sequence.

## 3. Algorithm

In this section, the algorithm for estimating the projection function and the coning angle is presented. First, the discrete emissivity-area product time sequence $Y={\left[Y\left(0\right),Y\left(1\right),\cdots ,Y\left(N-\;1\right)\right]}^{T}$ is extracted from the IR signature [16], where *N* is the number of the observing samples. Second, by fitting the emissivity-area product model proposed in this paper to the extracted discrete emissivity-area product time sequence, the model parameters are estimated.

For the parameter estimation, due to the fact that the coning period has been estimated in the radar community [8], we assume the coning angle speed *ω* is known. Now, the unknown parameters in the emissivity-area product model are the coefficient vector $x=\varepsilon \cdot s=\varepsilon \cdot {\left[a_{0},a_{1},\cdots ,a_{K},b_{0},b_{1},\cdots ,b_{K},c\right]}^{T}$ (see Equations (2) and (9)) and the motion and initial attitude parameters $m={\left[\alpha_{N},\beta_{N},\alpha_{0},\beta_{0}\right]}^{T}$. The least square error estimator is used to estimate these parameters, which is represented as

(14) $$\begin{array}{c} \left(\hat{m},\hat{x}\right)=\arg\min_{\left(m,x\right)}{∥Y-D_{m}x∥}_{2}^{2},s.t.\sum_{i=1}^{K+1}x_{i}-\sum_{i=K+2}^{2K+2}x_{i}=0,x_{i}\geq 0,i=1,2,\cdots ,2K+3 \end{array}$$

where the N×(2K+3) dimension matrix $D_{m}=\left[A_{0},\cdots ,A_{K},B_{0},\cdots ,B_{K},C\right]$ depends only on the parameter vector m, and not on the coefficient vector x. Given m, the observing angle sequence cosγ(n),n=0,1,⋯,N−1 is decided (see Equation (13)). Thus, base projection vectors $A_{i}={\left[A_{i}\left(\gamma \left(0\right)\right),A_{i}\left(\gamma \left(1\right)\right),\cdots ,A_{i}\left(\gamma \left(N-1\right)\right)\right]}^{T}$, $B_{i}={\left[B_{i}\left(\gamma \left(0\right)\right),B_{i}\left(\gamma \left(1\right)\right),\cdots ,B_{i}\left(\gamma \left(N-1\right)\right)\right]}^{T}$ and C=[C(γ(0)), C(γ(1)), ⋯, ${C\left(\gamma \left(N-1\right)\right)]}^{T}$ are also decided. Then, $D_{m}$ is decided. There are 2K+7 unknown parameters. In the experiments, K=50, so the dimension of unknown parameter space is 103. The optimization in Equation (14) is a non-linear problem, and a multi-start point iterative algorithm is needed to acquire the global minimum. However, the high dimension of unknown parameter space decreases the probability of selecting good seeds, which make the optimization a difficult problem. Luckily, there exists an approach in which the four non-linear parameters in m can be optimised separately from shape coefficients in x.

The particular parameter estimating algorithm is inspired from the form of the optimisation metric. If fixing m (the matrix $D_{m}$ is also fixed), the corresponding parameter vector x with the minimal square error can be decided by

(15) $$\hat{x}\left(m\right)=\arg\min_{x}{∥Y-D_{m}x∥}_{2}^{2},s.t.\sum_{i=1}^{K+1}x_{i}-\sum_{i=K+2}^{2K+2}x_{i}=0,x_{i}\geq 0,i=1,2,\cdots ,2K+3$$

This is just a convex optimization problem and there are standard tools for solving this problem. In this work, the $\hat{x}\left(m\right)$ is decided using the interior point method [20]. By replacing x with $\hat{x}\left(m\right)$, the minimum error corresponding to m is $∥Y-D_{m}\hat{x}{\left(m\right)∥}_{2}^{2}$, which is only a function of parameter m. Based on this fact, first, the estimate of m is acquired by optimizing the following problem

(16) $$\hat{m}=\arg\min_{m}{∥Y-D_{m}\hat{x}\left(m\right)∥}_{2}^{2}$$

In this work, this is implemented with the multiple starting point search algorithm in the Matlab global optimization toolbox.

**Figure 5:**
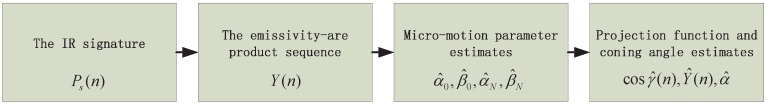
Process flow of the estimation scheme.

After the vector $\hat{m}$ has been acquired, the estimate of the cosine of the observing angle sequence $\cos\hat{\gamma }\left(n\right),n=0,1,\cdots$,N−1 is computed, and then $D_{\hat{m}}$ and the estimate of the projection coefficients $\hat{x}\left(\hat{m}\right)$. In addition, the estimate of the emissivity-area product sequence is computed as $\hat{Y}=D_{\hat{m}}\hat{x}\left(\hat{m}\right)$. Because the projection function is just the relation between the emissivity-area product and the observing angle, the estimation of the cosine observing angle and emissivity-area product represents the estimation of the projection function. The angle between the symmetric axis and the coning axis is just the coning angle, which is computed as

(17) $$\hat{\alpha }=\arccos(|\vec{e}\cdot \vec{n_{0}}\left|\right)$$

where $\vec{e}$ = ($\cos\alpha_{N}\cos\beta_{N},\sin\alpha_{N}\cos\beta_{N},\sin\beta_{N}$)${}^{T}$ denotes the unit direction vector the coning axis, while ${\vec{n}}_{0}$ = ($\cos\alpha_{0}\cos\beta_{0},\sin\alpha_{0}\cos\beta_{0},\sin\beta_{0}$)${}^{T}$ denotes the unit direction vector of the symmetric axis. Using the absolute value restricts the coning angle in the range ($0^{\circ },90^{\circ }$). Figure 5 shows the process flow of the proposed algorithm.

## 4. Experiments

### 4.1. Influence of Noise

In this section, some computer simulations and further discussion of their performance are conducted to verify the effectiveness of the proposed method for estimating the coning angle and the projection function.

The proposed estimating algorithm is tested on the simulated IR signature of the conical object. The observing interval is 10 s. The frame frequency of the IR sensor is 20 Hz. In the reference coordinate system, at the moment of 0 s, the azimuthal angle of line of sight is $0^{\circ }$ and the latitudinal angle is $90^{\circ }$. For simplicity, the azimuthal angle and latitudinal angle of line of sight are assumed to change linearly with the angle speed of $2^{\circ }$/s. The object is coning with period T=2 s. The half cone angle of the object is set as cotα=4. At the moment of 0 s, the azimuthal and latitudinal angle of coning axis are set as $0^{\circ }$ and $30^{\circ }$, respectively. The azimuthal and latitudinal angle of the symmetric axis are set as $0^{\circ }$ and $50^{\circ }$, respectively. Thus, the coning angle is $20^{\circ }$. The performance of the proposed method is tested under different signal noise ratios (SNRs). Monte Carlo simulations of 100 realizations are run for each SNR. The SNR varies from 0 dB to 20 dB with interval of 5 dB, which is calculated as $SNR=10\log_{10}\left(P_{s}/P_{n}\right)$, where $P_{s}$ is the power of the IR signature, and $P_{n}$ is the power of noise. The white Gaussian noise is added to the IR signature. For the projection representation, we set ${\left(\cot\alpha \right)}_{max}=5$ and Δ=0.01. Thus, the representation is a 103-dimensional vector.

**Figure 6:**
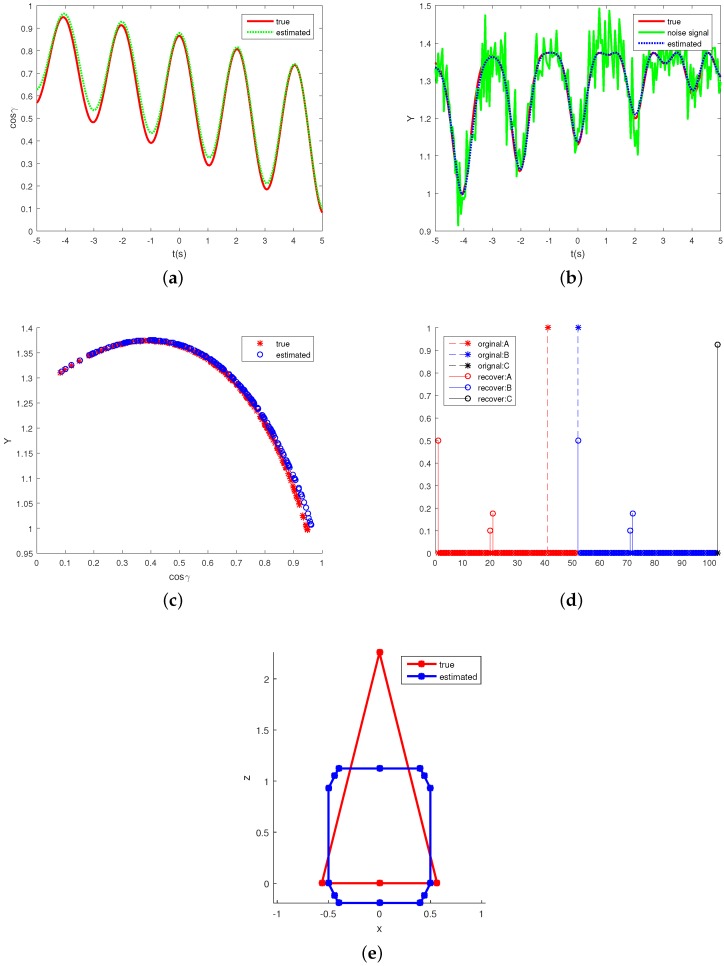
Estimation of the projecting functions. (**a**) shows the estimation of observing angles; (**b**) shows the estimating of the emissivity-area products; (**c**) plots the true and estimated cosine observing angle-emissivity-area product pairs, which also represent the projection functions; (**d**) illustrates the true and estimated projecting function representation, A denotes the coefficients of top cone base functions, B denotes coefficients of the bottom cone base function and C denotes the medium cylinder base function coefficient; and (**e**) shows the side view of the true and estimated shape.

Figure 6 compares some estimating results of the projection function with the true values in one Monte Carlo realization. Figure 6a shows the true and estimated observing angles (or negative estimated observing angles). The estimation of the emissivity-area product is depicted in Figure 6b. “True” in the figure denotes the theoretical values, “noise” denotes the signal extracted from the noise IR signature. In Figure 6c, the true function Y(cosγ) and the estimated function are illustrated. Figure 6d shows the true and the estimated projecting representation. From index 1 to 51 are the coefficients of the top cone base functions whose cotα ranges from 0 to 5 with step of 0.01, from 52 to 102 are the coefficients of bottom cone base functions and index 103 is the coefficient of the medium cylinder base function. The estimated shape representation gets non-zero top cone coefficients at cotα of 0, 1.9, 2, non-zero bottom cone coefficients also at cotα of 0, 1.9, 2 and non-zero medium cylinder coefficient. However, the true shape representation only gets a non-zero top cone coefficient at cotα of 4 and non-zero bottom cone coefficients also at cotα of 0. Figure 6e shows the side view of the true and recovered shapes. This shows that different shapes may generate similar projecting curves. Thus, instead of using the error between the true and estimated shape representation m to measure the projection estimating performance, the estimating error for the observing angle cosγ and the emissivity-area product *Y* are used to assess the performance of the algorithm. The projected area is a function of the observing angle. This means that if we can estimate the observing angle and the corresponding projected area, the projected function is also estimated.

As the performance measure of the estimation of the emissivity-area products, the MSE (mean square error) is used

(18) $$ERR\left(Y\right)=10\log_{10}\left[\frac{1}{M}\sum_{m=1}^{M}\frac{\sum_{i=0}^{N-1}{\left(Y\left(i\right)-{\hat{Y}}_{m}\left(i\right)\right)}^{2}}{\sum_{i=0}^{N-1}Y{\left(i\right)}^{2}}\right]$$

where Y(i),i=0,1,⋯,N−1 is the true emissivity-area product sequence, other than the one computed from the noise IR signature directly. ${\hat{Y}}_{m}\left(i\right),i=0,1,\cdots ,N-1$ is the estimated sequence in the *m*-th Monte Carlo simulation. *M* is the number of Monte Carlo realizations. The mean RMSE (root mean square error) is used to measure the estimating performance of the observing angle, which is computed as

(19) $$ERR\left(\cos\gamma \right)=\frac{1}{M}\sum_{m=1}^{M}\sqrt{\frac{1}{N}min(∥r-{\hat{r}}_{m}{∥}_{2}^{2},∥r+{\hat{r}}_{m}{∥}_{2}^{2})}$$

where $r={\left[\cos\gamma \left(0\right),\cos\gamma \left(1\right),\cdots ,\cos\gamma \left(N-1\right)\right]}^{T}$ are true observing angles, while ${\hat{r}}_{m}$ is the estimated coning angles in the m-th Monte Carlo simulation. The minimum operation is used because $A_{C}\left(\cos\gamma \right)$ is an even function. cosγ and −cosγ will generate the same projection. Thus, solutions similar to −r will also be reasonable. The RMSE is used to assess the estimating performance of coning angle, which is calculated as

(20) $$RMSE\left(\alpha \right)=\frac{100}{\alpha }\sqrt{\frac{1}{M}\sum_{m=1}^{M}{\left({\hat{\alpha }}_{m}-\alpha \right)}^{2}}$$

**Figure 7:**
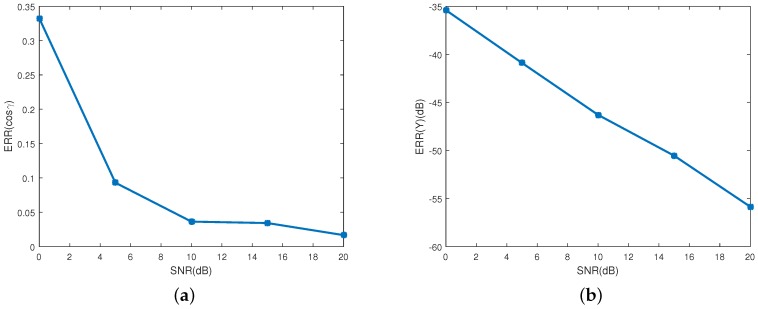
Estimating error of the projecting curve at different SNRs. (**a**) shows the estimating error of the observing angles; and (**b**) depicts the estimating error of the corresponding emissivity-area products.

**Figure 8:**
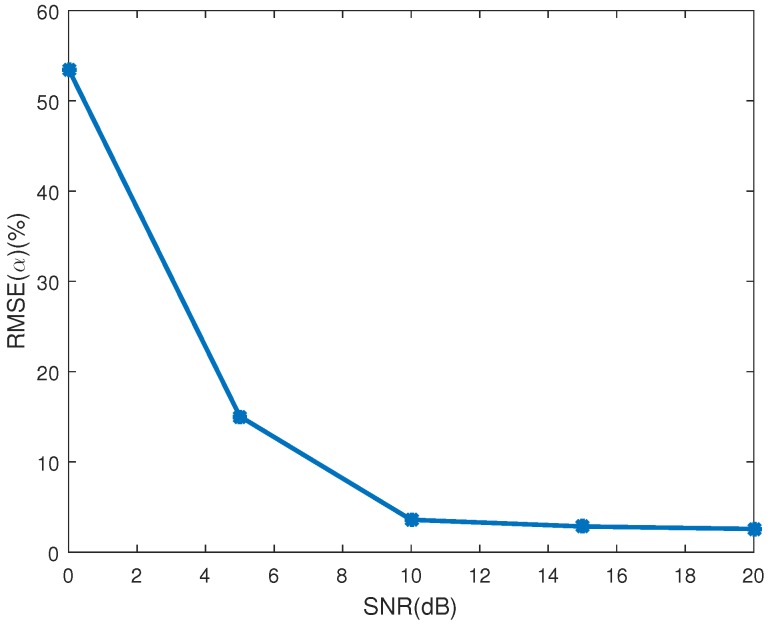
The estimating error of coning angle.

Figure 7a shows the ERR(cosγ) in different SNRs. As depicted in the figure, the estimating error decreases as the SNR increases. The mean estimating error of the emissivity-area product is showed in Figure 7b, in which the estimating performance increases as the SNR increases. When SNR is bigger than 10 dB, the estimating error of the emissivity-area product is less than −46 dB, and the estimating error of the observing angle is less than 0.036. The RMSE of the coning angle is showed in Figure 8. Similarly to the estimating performance of the observing angle and the emissivity-area product, the performance starts decreasing when the SNR is less than 10 dB. When the SNR is larger than 10 dB, estimating error is less than 3.6%.

### 4.2. Influence of the Estimating Error for Coning Period

In this work, we assume that the estimated coning period of the target is provided by the ground radar. However, inevitably, there will be estimating error for this parameter. The influence of this estimating error on the estimating performance of the proposed algorithm is studied in this section. As in [8], the estimating error of the micro-motion period is measured as

(21) $$ERR\left(T\right)=\frac{100}{T}\left|\hat{T}-T\right|$$

where *T* denotes the true coning period, and $\hat{T}$ denotes the estimated coning period. In this experiment, ERR(T) varies from 0% to 5% with interval of 1%. We also assess the performance of the algorithm at SNRs of 10, 15, and 20 dB. Monte Carlo simulations of 100 realizations are run for each SNR. Other parameters are set to be the same as Section 4.1.

**Figure 9:**
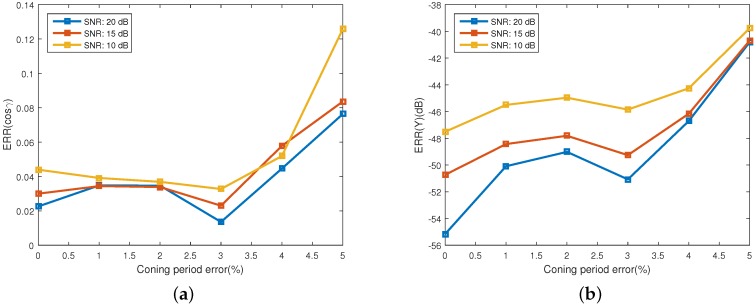
Estimating error of the projecting feature at different error of the estimated coning period. (**a**) shows the estimating error of the observing angles; and (**b**) depicts the estimating error of the corresponding emissivity-area products.

**Figure 10:**
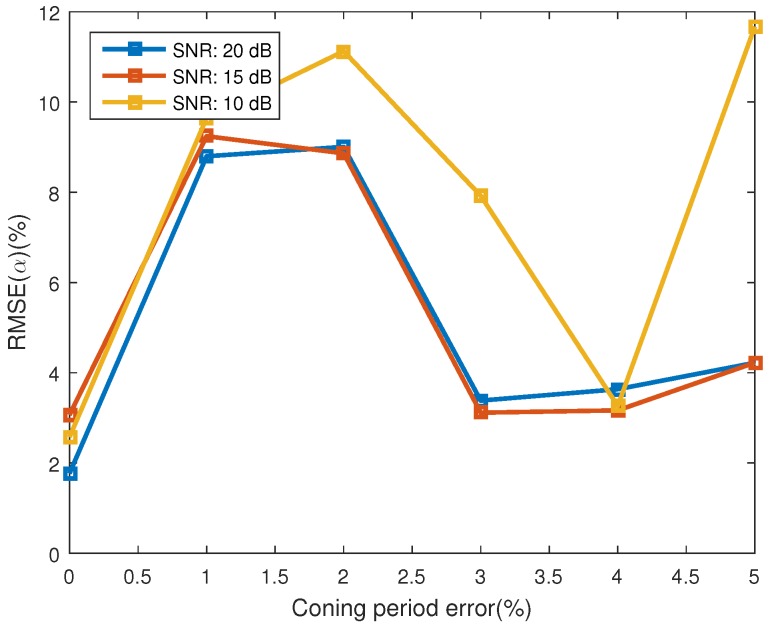
The estimating error of coning angle.

Figure 9 shows the estimating error for the projecting feature, which is represented with the estimating error of observing angles and the emissivity-area product. The error of observing angles is showed in Figure 9a and the error of the emissivity-area product is illustrated in Figure 9b. One interesting phenomenon is that the estimating performance of the emissivity-area product and the observing angle does not decrease consistently with the increase of coning period error. The performance has a local minimum at the coning period error of 3%. As depicted in Figure 10, similarly, the estimating performance of the coning angle also increases at the coning period error of 2% and decreases at 4%. The reason of this phenomenon is still unknown. When the coning period error is less than 4% and the SNR is bigger than 10 dB, the estimating error of the observing angle is less than 0.06 and the error of the emissivity-area product is less than −44 dB. In addition, the estimating error of the coning angle is less than 11.2%. In [21], the estimating error of the coning angle is less than 25%, which is also acquired at the coning angle of $20^{\circ }$. However, they used the radar data to estimate the coning angle.

### 4.3. Influence of Coning Angle

In this section, the algorithm is evaluated at different coning angles, which ranges from $0^{\circ }$ to $30^{\circ }$ with step of $5^{\circ }$. For each coning angle, the performance of SNRs ranging from 0 dB to 40 dB with step of 5 dB is studied. Monte Carlo simulations of 100 realizations are run for each SNR. For assessing the estimation accuracy of coning angle, the indicator of Equation (20) is not used since, when the coning angle is $0^{\circ }$, the indicator cannot be used. Instead, we use

(22) $$RMSE\left(\alpha \right)=\sqrt{\frac{1}{M}\sum_{m=1}^{M}{\left({\hat{\alpha }}_{m}-\alpha \right)}^{2}}$$

**Figure 11:**
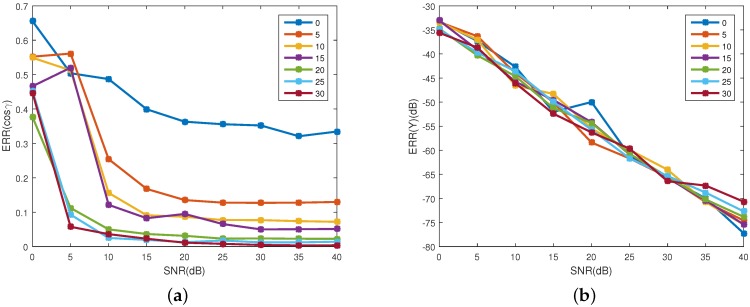
Estimating error of the projecting feature at different coning angles. (**a**) shows the estimating error of the observing angles; and (**b**) depicts the estimating error of the corresponding emissivity-area products. Different colors represent different coning angles, which range from $0^{\circ }$ to $30^{\circ }$ with step of $5^{\circ }$.

**Figure 12:**
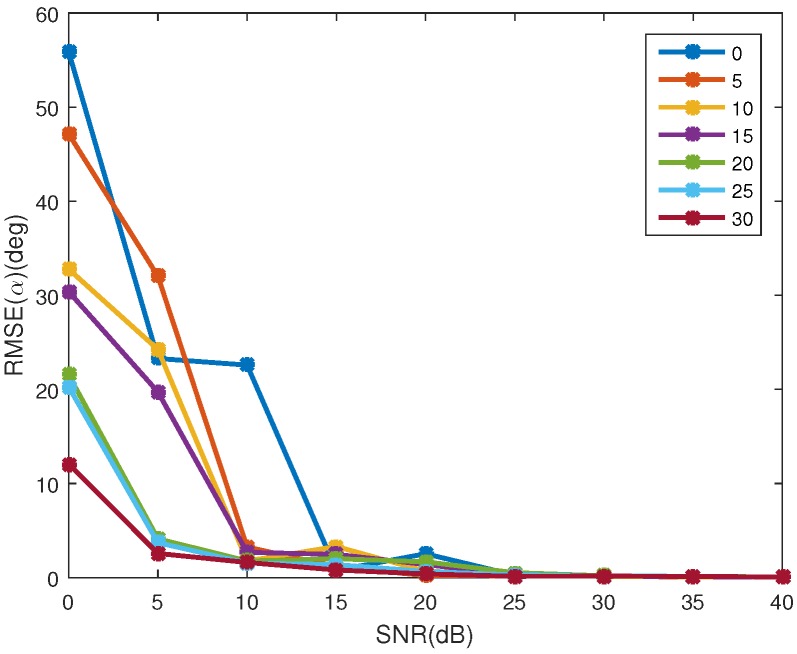
The estimating error of coning angle at different coning angles. Different colors represent different coning angles, which range from $0^{\circ }$ to $30^{\circ }$ with step of $5^{\circ }$.

Other parameters are set to be the same as Section 4.1. Figure 11 shows the estimation accuracy of the projection function. In Figure 11a, it is clear that with the increase of SNR, the error of the observing angle decreases for all coning angles, and the smaller the coning angle, the bigger the estimating error. When SNR>15 dB and the coning angle is bigger than $5^{\circ }$, the error of observing angle is less than 0.1. However, for the coning angle of $0^{\circ }$ (no coning micro-motion), the error is bigger than 0.3 for all the SNRs. Figure 11b shows the error of the emissivity-area products. With the increase of the SNR, the error decreases, and for all the coning angles, the error is similar. When SNR>10 dB, the error for all the coning angles is less than 40 dB. Thus, for the estimation of the projection function, the smaller the coning angle, the bigger the estimation error of the projection function, and coning micro-motion is important for the estimation of the projection function. Absence of the coning micro-motion leads to being unable to estimate the projection function. Figure 12 shows the estimation error of the coning angle. Different from the estimation of the projection function, the decrease of the coning angle does not increase the estimation error of coning angle. When SNR>15 dB, for all the given coning angles, the estimation error is less than $3.5^{\circ }$.

### 4.4. Influence of Reflected Energy

The signal model used in this paper only considers the radiation emitted by the target. In fact, the sensor also received radiation reflected by the target. In this section, the influence of the reflected energy is studied. As indicated in [19], the reflected energy mainly consists of infrared radiation from the sun and the earth, and reflected infrared radiation from the earth and atmosphere. The sensor’s wave band is 6∼12 μm. The band irradiance from the sun at the target is about 1.3 W/m${}^{2}$. The mean temperature of target is 300 K. The band radiation emitted by the black body is 120 W/m${}^{2}$. Thus, the reflected energy is far smaller than the emitted energy. According to the energy conservation law, there will be reflection, absorption and transmission when external radiation is transmitted to the target surface, and the total of energy percentage is 1, that is

(23) ρ(λ)+α(λ)+τ(λ)=1

where ρ(λ) is spectral reflectivity, α(λ) is the spectral absorptivity and τ(λ) is the spectral tansmissivity. Based on Kirchhoff’s law, the spectral emissivity ε(λ) is equal to its spectral absorptivity α(λ). In this work, we set τ(λ)=0. Thus, ρ(λ)+ε(λ)=1. The algorithm is tested with different emissivity to change the rate of the emitted and reflected energy in the received radiation. The emissivity varies from 0.5 to 1 with step of 0.1. For each emissivity, the SNR varies from 0 dB to 30 dB with step of 5 dB. In addition, Monte Carlo simulations of 100 realizations are run for each SNR. Equation (22) is used assess the estimation accuracy of the coning angle. Other parameters are set to be the same as Section 4.1.

**Figure 13:**
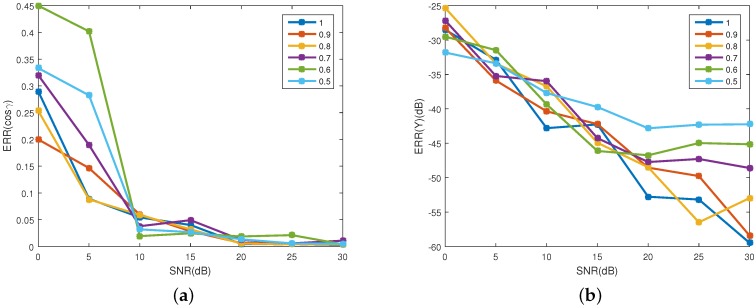
Estimating error of the projecting feature at different emissivity. (**a**) shows the estimating error of the observing angles; and (**b**) depicts the estimating error of the corresponding emissivity-area products. Different colors represent different emissivity, which ranges from 0.5 to 1 with a step of 0.1.

**Figure 14:**
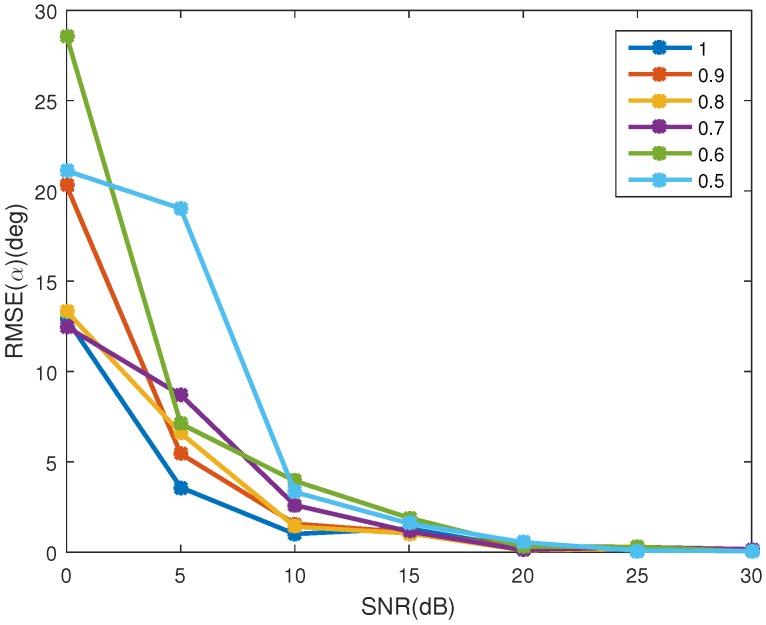
The estimating error of coning angle at different emissivity. Different colors represent different emissivity, which ranges from 0.5 to 1 with a step of 0.1.

Figure 13 shows the estimation accuracy of the projection function. In Figure 13a, with the increase of SNR, the error of the observing angle decreases for all the emissivity. When SNR>10 dB, the error of observing angle is less than 0.06. Figure 13b shows the error of the emissivity-area products. With the increase of the SNR, the error decreases. When SNR>10 dB, the error for all the coning angles is less than 35 dB. In addition, with the increase of SNR, the bigger the emissivity, the higher the estimation accuracy of the emissivity-area products. It can be concluded that when SNR>10 dB, despite the existence of the reflected radiation, the algorithm is effective for estimating the projection function. Figure 14 shows the estimation error of the coning angle. Similar to the case in the projection function estimation, with the increase of SNR, the estimation error decreases. In addition, when SNR>10 dB, the difference between the error of different emissivities is small. For all emissivity, the estimation error is less than $5^{\circ }$ when SNR>15 dB. In addition, when SNR>10 dB, the error is less than $2^{\circ }$.

### 4.5. Influence of Imaging

In the previous experiments, the received power is used as the input data directly while the imaging process is not considered. In reality, the non-sensitive bands between pixels cause only part of the energy to be acquired by the sensor. In this section, the influence of the non-sensitive bands is studied. Table 1 shows the parameters of the IR camera. In each frame, the amplitude of the IR radiance is computed by summing the response of the pixels in a 5 × 5 window centered at the position of the target. The algorithm is evaluated at different fill factors, which is defined as the ratio of a pixel’s light sensitive area to its total area and ranges from to 0.8 to 1 with a step of 0.05. The algorithm was also evaluated at different SNRs, ranging from 15 to 45 dB with a step of 5 dB. The noise is added to the response of pixels. Equation (22) is used assess the estimation accuracy of the coning angle. Other parameters are set to be the same as Section 4.1.

**Figure 15:**
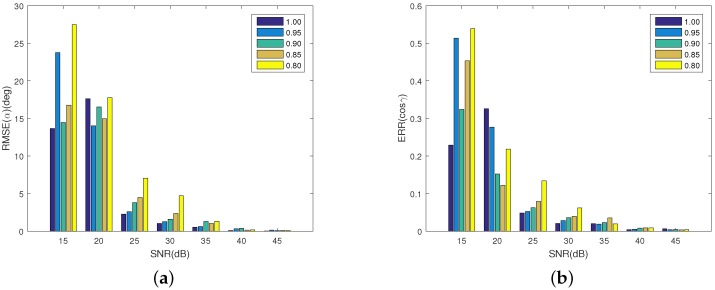
Estimating error of precession angle and observing angles. (**a**) shows the estimating error of the precession angle; and (**b**) depicts the estimating error of the observing angles. Different colors denote different fill factors.

Figure 15 shows the experiment results. Because the recovered amplitude of the IR signature is only part of the received radiance, the estimated emissivity-area products are smaller than the real values. Equation (18) cannot be used to assess the estimating accuracy of the projection function. Thus, only the estimating performance of the observing angle and precession are illustrated. In the case of the precession angle, when SNR is higher than 30 dB, the estimating errors at different fill factors are less than $1.4^{\circ }$. From 30 to 25 dB a decrease of the performance is observed, while less than 25 dB shows that the precession angle cannot be estimated. At 25 and 30 dB, the error increases as the fill factor decreases. At 30 dB, when the fill factor is bigger than 0.85, the error is less than $2.4^{\circ }$, while at 25 dB, when the fill factor is bigger than 0.9, the error is less than $3.8^{\circ }$. In the case of the observing angle in Figure 15b, the situation is similar. When SNR>30 dB, the error is less than 0.04. At 30 dB, the error is less than 0.065. At 25 dB, when the fill factor is bigger than 0.9, the error is less than 0.065.

## 5. Conclusions

This work analyses the time variation of an emissivity-area product that can be extracted from the IR signature and is proportional to the projection area. It is found that the changing of projection area is decided by the changing of observing angle and the projection function of the object. We show that the projection function of any rotationally symmetric object can be approximately represented as the linear combination of base projecting functions. Based on this linear projection function representation, the least square error estimator is used to estimate the direction of the symmetric axis of the object and the coning axis from the extracted emissivity-area product sequence. Then, estimates of the coning angle, the emissivity-area product, the observing angle and the projection coefficients can be acquired. Experiments validate the effectiveness of the proposed method. Further research on how to classify space objects based on the extracted micro-motion and shape features will be carried out.

## Figures and Tables

**Table 1:** Parameter setting of infrared (IR) camera.

Resolution (pixel)	128 × 128	Pixel size (μm)	30 × 30
Focal length (mm)	100	Optical aperture (cm)	10
Wavelength range (μm)	8∼12	Diffusion coefficient $\sigma_{psf}$ (pixel)	0.5
